# A Critical Appraisal of Extracorporeal Photopheresis as a Treatment Modality for Acute and Chronic Graft-Versus-Host Disease

**DOI:** 10.3390/biomedicines5040060

**Published:** 2017-10-11

**Authors:** Hind Rafei, Mohamed A. Kharfan-Dabaja, Taiga Nishihori

**Affiliations:** 1 Department of Internal Medicine, George Washington University School of Medicine, Washington, DC 20052, USA; hindrafei@email.gwu.edu; 2 Department of Blood & Marrow Transplantation and Cellular Immunotherapy, H. Lee Moffitt Cancer Center and Research Institute, 12902 Magnolia Drive, Tampa, FL 33612, USA; Mohamed.Kharfan-Dabaja@moffitt.org

**Keywords:** extracorporeal photopheresis, steroid-refractory acute and chronic graft-versus-host disease

## Abstract

Although significant advances have been made in the biologic understanding of graft-versus-host disease (GVHD) and its treatment options, GVHD remains the single most challenging obstacle to the success of allogeneic hematopoietic cell transplantation (HCT) due to high risk of disabling morbidity and mortality. Extracorporeal photopheresis (ECP) has promising effects in controlling steroid-refractory GVHD, both acute and chronic, and it has been studied extensively. Its putative immunomodulatory mechanisms, while not immunosuppressive, position ECP as an attractive treatment strategy for GVHD patients who are already receiving global immunosuppression. However, ECP is relatively underutilized due in part to limited access and time commitment. Here, we review the recent findings on the ECP efficacy in both acute and chronic GVHD, primarily for steroid-refractory status, and we critically appraise its benefits. We also explore salient considerations on the optimal use of ECP in the treatment of refractory GVHD.

## 1. Introduction

Allogeneic hematopoietic cell transplantation (HCT) remains the only curative treatment option for many patients with various malignant and benign hematologic disorders [1]. With the increasing utilization of allogeneic HCT in part due to the introduction of reduced intensity conditioning regimens, expansion of HCT to higher recipient age, increasing use of alternative donors and filgrastim-mobilized peripheral blood stem cells [2] among others, the overall burden of patients surviving with graft-versus-host disease (GVHD) may also be growing worldwide [3]. Despite improvements in pharmacologic prevention and treatment of GVHD, graft manipulation technologies, and advances in supportive care, GVHD continues to be the major source of disabling morbidity and mortality after allogeneic HCT limiting its broader application.

Pathophysiology of GVHD is complex and beyond the scope of this review; however, acute GVHD is mediated by tissue injury from preparative regimen, cytokines and alloreactive cytotoxic T cell responses, and chronic GVHD results from complex dysregulation of both adaptive and innate immune responses, resulting in a variety of autoimmune-like conditions. Corticosteroid remains the established standard first line therapy for GVHD, and multiple second-line therapies have been explored to regain responses in those patients with steroid-refractory or steroid-intolerant GVHD [4]. Major obstacles of GVHD treatments are rather limited responses to treatments, long-term steroid toxicities, and global immunosuppression with increased risk of infections, morbidity and ultimate mortality. Extracorporeal photopheresis (ECP) is an attractive option for GVHD patients based on its immunomodulatory properties and promising responses. Here, we review and critically appraise the current literature on ECP as a treatment option for patients with acute and chronic GVHD.

## 2. Extracorporeal Photopheresis (ECP)

ECP exerts its immunomodulatory properties by extracorporeal exposure of peripheral blood mononuclear cells to 8-methoxypsolaren and ultraviolet (UV) A light in an apheresis procedure and subsequent reinfusion of the treated cells back into the patient’s circulation [5]. ECP has been utilized for GVHD treatment since the 1980s [6] but its only FDA-approved indication remains for the treatment of cutaneous T cell lymphoma. Proposed immunomodulatory properties of ECP may be derived from the following mechanisms: differentiation of monocytes into dendritic cells (DCs), apoptosis of cytotoxic cells, and/or impaired antigen presentation capacity. Additionally, studies have also shown increased natural killer (NK) cells, modulation of DCs, and skewing toward Th2 response [7,8]. While clinical benefits of ECP treatment have been well documented, its exact mechanisms of action remain rather unclear.

## 3. ECP as a Treatment for Acute Graft-Versus-Host Disease (GVHD)

### 3.1. Prospective Studies

In a study of 59 patients with acute steroid-refractory GVHD (grades II to IV) treated with ECP given on two consecutive days at 1 to 2-week intervals until improvement and then every 2 to 4 weeks until maximum response, Greinix et al. noted complete response (CR) rate of 82% in cutaneous GVHD, 61% in liver GVHD, and 61% in gut GVHD [9]. The probability of survival was 59% among complete responders compared to 11% in patients with incomplete responses [9]. Overall survival (OS) at 4 years was significantly better in patients with CR compared to those who did not achieve CR (59% versus 11%, *p* < 0.0001) [9]. Intensified ECP (2–3 treatments per week on a weekly basis) was found to be significantly efficacious with improved CR rates in patients with GI involvement (73% versus 25%) and those with grade IV GVHD (60% versus 12%) [9].

Using an adaptive Bayesian design, Alousi et al. conducted a phase II, randomized study of 81 newly diagnosed acute GVHD patients who received <72 h of steroids, and they were randomized to receive 2 mg/kg of methylprednisolone with (*n* = 51) or without ECP (*n* = 30) [10]. ECP was delivered for 8 sessions during days 1–14, 6 sessions during days 15–28, and then 8 sessions during days 29–56 [10]. ECP was found to be more efficacious in skin only acute GVHD (72% vs. 57% response rate) but visceral-organ involvement response rates were similar [10]. By day 56, 43% of the patients in the ECP arm were on physiologic doses of steroids versus 30% in the control arm (*p* = 0.34) [10]. ECP was associated with more robust recovery of CD4^+^ and CD8^+^ cells and higher number of regulatory T-cells [10]. Results of prospective study of ECP in acute GVHD are summarized in Table 1.

### 3.2. Retrospective Studies

Several reports regarding the efficacy of ECP in acute GVHD are available. In a single-center study of 27 patients with steroid-resistant GVHD who received ECP (for 6 courses in the first 3 weeks followed by cessation if patients achieved CR or weekly maintenance therapy until CR), Garban et al. found that 9 out of 12 patients with acute GVHD responded to treatment [11]. There appeared to be no response in those with liver GVHD. The authors concluded that ECP yielded the most benefit if performed as early as possible following the diagnosis of acute GVHD [11]. Perfetti et al. reported 23 patients with steroid-refractory acute GVHD who were treated with ECP and 52% of patients achieved CR [12]. CR was achieved in 70%, 42% and 0% of grades II, III, and IV acute GVHD, respectively [12]. CR was achieved in 66%, 40% and 27% of skin, gut and liver GVHD, respectively. There was a suggestion that patients treated within 35 days of onset of acute GVHD may have higher responses (83% versus 47%, *p* = 0.1) [12].

A retrospective multicenter comparative analysis of ECP versus anticytokine therapy (inolimomab or etanercept) as a second-line treatment for steroid-refractory acute GVHD were reported by Jagasia et al. [13]. Both overall response rate (ORR) and CR rate were higher in the ECP group (66% versus 32%, *p* = 0.001; 54% versus 20%, *p* = 0.001, respectively) [13]. In multivariate analyses, ECP, adjusted for conditioning regimen intensity and steroid dose, was associated with superior survival (hazard radio (HR) 4.6, *p* = 0.016) [13]. ECP schedules were not uniform between the groups and those in the anti-cytokine cohort had a higher proportion of patient receiving T-cell replete grafts, stage 3–4 skin GVHD and receiving steroid at a dose ≥2 mg/kg [13]. Table 1 summarizes the outcomes of 3 representative retrospective studies.

### 3.3. Systematic Reviews

Abu-Dalle et al. conducted a systematic review of 9 prospective studies (1 randomized controlled trial and 8 single-arm studies) for both acute and chronic GVHD treatment with ECP and reported pooled analyses of 54 subjects (6 studies) with acute GVHD [14] (Table 1). ORR for acute GVHD was 69% (95% confidence interval (CI): 34–95%). There was high heterogeneity between studies. Pooled ORR for specific organs showed cutaneous 84% (95% CI: 75–92%), GI 65% (95% CI: 52–78%), and hepatic 55% (95% CI: 35–74%) [14]. Rate of immunosuppression discontinuation was 55% (95% CI: 40–70%) [14].

### 3.4. Consensus Statements, Guidelines and Recommendations

The American Society of Blood and Marrow Transplantation (ASBMT) developed recommendations on the second-line systemic treatment of acute GVHD based on results of 29 studies (including 2 studies on ECP) which evaluated various treatment modalities for acute GVHD [4,12,15]. Based on the evaluation of 6-month survival estimates by ASBMT, no specific second-line modality was recommend over other options, however, ECP was listed as a potential second-line choice for acute GVHD treatment [4]. The recommended ECP treatment schedule is 3 times per week (week 1), 2 times per week (weeks 2–12) and 2 times every 4 weeks thereafter [4]. A joint working group established by the British Committee for Standards in Haematology (BCSH) and the British Society for Bone Marrow Transplant (BSBMT) recommends ECP as a potential second line treatment for acute grade III-IV GVHD if no improvement after 5 days or progression within 72 h of 2 mg/kg of methylprednisolone (grade 2c recommendation) [16].

The Italian Society of Hemapheresis and Cell Manipulation (SIdEM) and the Italian Group for Bone Marrow Transplantation (GITMO) reviewed 11 published reports on 293 patients and recommended the use of ECP for acute GVHD not responding to steroid and calcineurin inhibitors [17]. Better results are anticipated in patients with isolated skin involvement and the efficacy of ECP in visceral-organ GVHD is less well established [17]. The European Dermatology Forum (EDF) recommends the use of ECP as a second line in patients with acute GVHD not responding to first-line steroid therapy at 2 mg/kg/day (defined as progression of acute GVHD after 3 or more days or lack of response after 7 or more days of steroids) [18]. Treatment schedule recommended is twice or three times per week until CR [18].

UK and Scandinavian Photopheresis Expert Group reviewed and summarized currently published guidelines [19]. In their consensus statement, they recommend ECP to be considered as second-line therapy for patients with acute grade II-IV GVHD who are either steroid refractory (worsening of acute GVHD after 3 days of corticosteroids (minimal dose of 1 mg/kg) or no improvement after 7 days of corticosteroids (minimum dose of 1 mg/kg), steroid dependent (recurrence of acute GVHD (grade II or higher) during corticosteroid taper and before reaching 50% of initial starting dose of corticosteroids) or steroid intolerant (acute GVHD unable to tolerate the side effects of adequate doses of corticosteroids) [20]. They recommend 2 consecutive days of ECP treatment weekly for a minimum of 8 weeks [20]. Additionally, patients with grade III-IV acute GVHD may benefit from three treatments per week for the first 4 weeks [20]. ECP was also thought to be a valuable option for immunosuppressed patients who are unable to tolerate systemic steroids or other immunosuppressive second-line agents. Adult patients who achieve CR and receiving <20 mg of methylprednisolone or equivalent (<0.5 mg/kg for pediatric patients) may be able to stop ECP after 8 weeks of therapy [20].

Based on the available data, authors recommend considering ECP as second line therapy for acute GVHD patients who are steroid refractory or dependent. ECP should be started at minimum of 2 days per week (ideally 3 days per week the first week: intensified regimen) and be continued for 2 to 3 months on a weekly basis, or until the resolution of GVHD symptoms. ECP would also be helpful in those patients who may not be able to tolerate systemic steroids.

## 4. ECP as Treatment for Chronic GVHD

### 4.1. Prospective Studies

Reports on the efficacy of ECP for the treatment of chronic GVHD date back to the early 1990s when several case reports and small studies demonstrated encouraging results [19,21,22,23,24,25]. Following these early reports, numerous studies were conducted to evaluate the efficacy of ECP in chronic GVHD with overall encouraging results though generalizability of these findings are limited. The best evidence comes from a randomized controlled trial by Flowers et al. in 2008 where ECP as the treatment of chronic GVHD was evaluated in 95 patients with cutaneous chronic GVHD that was not adequately controlled with steroid alone [26]. This multicenter study randomized steroid-dependent, refractory or intolerant chronic GVHD patients to either ECP (3 times weekly during week 1, twice weekly on consecutive days weeks 2–12, then 2 ECP treatments every 4 weeks until week 24 in responding patients) in addition to standard immunosuppression (*n* = 48) or immunosuppression alone (*n* = 47) [26]. The study failed to meet the primary endpoint of blinded quantitative comparison of median change from baseline on total skin score (TSS) over 10 body regions at 12 weeks (14.5% improvement in ECP arm vs. 8.5% in control arm, *p* = 0.48) [26]. However, ECP showed a corticosteroid-sparing benefit as well as investigator-assessed response with good tolerance [26]. In the follow-up study, 29 patients who were originally assigned to the controlled arm were allowed to cross-over, and received ECP treatment [27]. Clinical benefits were noted, but they were at slightly reduced response rates compared to the other studies (31% with complete or partial skin response) [27]. The highest extracutaneous chronic GVHD response was seen in oral mucosa (70% complete or partial response), and 33% had more than 50% reduction in corticosteroid dose at the end of 24-week treatment [27].

In a prospective study of 25 patients with extensive, steroid-refractory chronic GVHD treated with ECP (2 consecutive days every 2 weeks in 17 patients and once a week in 8 patients) by Foss et al. [28], 20 patients had improvement in cutaneous GVHD while only one patient with lung involvement had a 50% improvement in carbon monoxide diffusion (DLCO) and one with biopsy-proven GVHD of the gut had resolution of diarrhea [28]. ORR was 64% [28]. Seaton et al. reported 28 patients with advanced chronic GVHD treated with ECP (2 consecutive days every 2 weeks for 4 months and then monthly), and 38% of evaluable patients with cutaneous disease responded at 3 months with 48% response rate at 6 months [29]. For other organs involved with GVHD, a nonsignificant improvement in liver function tests occurred (at least 25% improvement) and there was no improvement in the pulmonary or neuromuscular function [29]. These studies highlight the differential, organ-specificeffects of ECP on chronic GVHD. Table 2 summarizes the results of 4 prospective studies and organ-specific chronic GVHD response rates.

### 4.2. Retrospective Studies

There have been multiple retrospective studies on ECP treatment for chronic GVHD [11,30]. We highlight some representative studies in our review. Couriel et al. retrospectively evaluated 71 patients with severe chronic steroid-refractory GVHD treated with ECP [31]. ORR was 61% including 20% achieving a CR. The best responses were achieved in chronic GVHD affecting the oral mucosa (77%), liver (71%), eyes (67%), skin (59%) and lungs (54%) [31]. There was also evidence of a corticosteroid-sparing effect with a cumulative incidence of steroid discontinuation at 22% (at 1 year) [31]. Dignan et al. reported 82 refractory mucocutaneous chronic GVHD patients treated with bi-monthly ECP for 6 months with a subsequent taper to monthly regimen on response [32]. ORR was 79%, and 77% of patients who completed 6 months of ECP had a reduction in immunosuppressive dose [32]. In a large retrospectively study, Del Fante et al. analyzed 102 chronic GVHD patients treated with ECP over 14-year period and reported ORR of 80.4% [33]. Univariate analysis showed lung GVHD as a predictor of poor response to ECP (hazard ratio 0.34; 95% CI: 0.12–0.94, *p* = 0.038) [33]. Three large-scale retrospective study results are summarized in Table 2.

### 4.3. Pooled Results from Systematic Reviews and Meta-Analyses

As noted previously, Abu-Dalle et al. conducted a systematic review and reported pooled analyses of 87 subjects (5 studies) with chronic GVHD [14]. ORR for chronic GVHD was 64% (95% CI: 47–79%). Though heterogeneity was high, pooled ORR for specific organs showed cutaneous 71% (95% CI: 57–84%), GI 62% (95% CI: 21–94%), hepatic 58% (95% CI: 27–86%), oral mucosa 63% (95% CI: 43–81%), musculoskeletal 45% (95% CI: 18–74%), and pulmonary 15% (95% CI: 0–50%) [14]. Pooled incidence of any grade 3 or 4 adverse events were 38% (95% CI: 6–78%) [14]. ECP-related mortality was low with one death in one study (attributed to sepsis and idiopathic respiratory distress syndrome) out of 4 studies [14]. The pooled rate of immunosuppression discontinuation was 23% (95% CI: 7–44%) [14].

A systematic review and meta-analysis of 18 studies (both prospective and retrospective) evaluating the efficacy of ECP in steroid-refractory chronic GVHD with 595 patients was conducted by Malik et al. [34]. The pooled ORR on different organs for chronic GVHD treated with ECP were skin 74% (95% CI: 60–85%), liver 68% (95% CI: 57–77%), ocular 60% (95% CI: 40–78%), oral 72% (95% CI: 51–86%), lung 48% (95% CI: 33–63%), GI 53% (95% CI: 21–83%), and musculoskeletal 64% (95% CI: 18–94%) [34] (Table 2). When retrospective studies are included, lung response appears to be much higher than those with only prospective studies, perhaps highlighting an inherent bias associated with retrospective attribution of responses.

### 4.4. Consensus Statements and Clinical Practice Guidelines

Some groups have published consensus statements on the use of ECP for the treatment of chronic GVHD. The SIdEM and the GITMO jointly developed recommendations on the appropriate applications of ECP in patients with GVHD [17]. During the process, they also reviewed 23 studies (with a minimum of 11 patients per study) reporting the outcomes of ECP in 735 chronic GVHD patients [17]. The ORR and CR rate were 64% and 3% in cutaneous GVHD, 56% and 27% in hepatic GVHD, respectively [17]. The ORR was 47–57% in oral mucosa and GI GVHD. High response rates were also observed in children with ocular involvement [17].

UK and Scandinavian Photopheresis Expert Group summarized 23 studies on chronic GVHD treated with ECP in 521 patients (up to 2005) and reported that based on the 18 studies reporting the responses, the mean cutaneous chronic GVHD response rate was 68% (range, 29–100%) while that of hepatic chronic GVHD (reported in 10 studies) was 63% and 63% for mucosal GVHD (9 studies) [35]. The 2017 update by the same group reviewed 27 studies with 725 patients treated with ECP for chronic GVHD and showed mean ORR of 68% (14 studies) and the following individual organ mean response rates: cutaneous GVHD 74% (23 studies), hepatic GVHD 62% (15 studies), ocular GVHD 60% (4 studies), mucosal GVHD 62% (12 studies), GI GVHD 46% (5 studies), and pulmonary GVHD 46% [19]. Chronic GVHD organ-specific responses derived from these consensus statement studies are summarized in Table 2.

A joint working group established by the BCSH and the BSBMT recommends that ECP may be considered as a second-line treatment in skin, oral, or liver chronic GVHD [36]. ECP schedule should be every 2 weeks treatments for a minimum assessment period of 3 months (grade 1 recommendation: evidence of benefits and no other immunosuppressive therapy received a stronger recommendation for second-line treatment of chronic GVHD) [36]. ECP may be considered as a third-line option for chronic GVHD involving other organs (such as GI and lung) which was a grade 2 recommendation (judicious application to individual patients is required) [36]. The German/Austrian/Swiss consensus conference on clinical practice in chronic GVHD recommended ECP in second-line treatment (C-1 grading: use in second-line treatment was justified) [37,38]. The two Italian societies (SIdEM and GITMO) recommend ECP in both adults and pediatric patients with chronic GVHD that is steroid-resistant or steroid-dependent, irrespective of disease extent and severity [17]. They consider ECP a valuable option with the potential for steroid sparing in responsive patients [17].

Authors believe that ECP would be a viable second- or third-line treatment option for chronic GVHD patients where ECP can be performed every 2 weeks for approximately 3 months to assess initial response. ECP has favorable response rates in the majority of chronic GVHD patients (ORR > 60%) with higher organ specific response rates in cutaneous, oral mucosa, ocular and GI tract.

## 5. Discussion

ECP plays a pivotal role mostly as a second-line therapy for both acute and chronic steroid-refractory GVHD. Its steroid-sparing benefit and facilitation of steroid taper have been demonstrated in multiple studies, and it remains an attractive option especially for those who cannot tolerate corticosteroids [28,32,39,40,41]. A large number of investigators have reported the efficacy of ECP in treating steroid-refractory GVHD with varying success rates and some studies have demonstrated survival benefits with ECP [28,31,33]. A variety of ECP treatment schedules have been recommended but in general, intensive short courses of ECP may be considered in acute GVHD whereas longer treatment could be necessary in chronic GVHD. In terms of the organ specific responses, cutaneous GVHD seems to have the highest response rates and lung (chronic GVHD) on the other hand may have rather disappointing results.

Although numerous studies on ECP, including those with open label randomized designs, are available, the quality of evidence on the ECP as a treatment option for GVHD is somewhat limited in part due to the absence of blind studies of ECP. Many of the studies quoted in the recommendations are also retrospective in nature. Acknowledging the limitations of clinical evidence which supports the use of ECP, the procedure has benefits to GVHD patients not only based on its therapeutic efficacy but also based on its well-established safety profile. Of more than 500,000 ECP treatments performed worldwide since 1987, the incidence of reported adverse events is less than 0.003% [19]. The most commonly reported side effects are mild and include nausea, fever and headache. On the other hand, significant reactions such as vasovagal syncope or infections secondary to indwelling catheters are infrequent [19,35,36,42,43].

Though ECP is generally safe, a few considerations are worth noting. First, the ECP circuit has a relatively small fluid volume (typically < 500 mL). ECP would be best performed in a closely monitored outpatient unit or in the hospital, and one has to be mindful of the volume status of potentially ill GVHD patients. Secondly, intravenous 8-methoxypsolaren is only licensed in the Therakos closed system, and those patients who are photosensitive or have a sensitivity to psolaren compounds are excluded from ECP option. Additionally, weight under 40 kg is contraindicated in the ECP Therakos UVAR-XTS^TM^ machine as low body mass patients would unlikely be able to tolerate the volume shifts during ECP treatment. However, the closed system CELLEX^TM^ (Therakos) has recently replaced the UVAR-XTS^TM^ (Therakos) system. The CELLEX^TM^ (Therakos) system allows shorter treatment time (down to one and a half hour from typical three-hour treatment) and treatment of lower body weight patients expanding the ECP indications to pediatric population [19]. Both heparin or citrate can be fused to prevent thrombosis during the ECP but citrate may be the preferred method of anticoagulation in patients with risk of bleeding from especially GVHD (such as GI or liver). A low platelet count (such as <20 × 10^9^/L) is considered a contraindication to ECP. Additionally, patients would need to have appropriate venous access such as photopheresis-compatible port-a-cath or central venous catheter. Physicians should discuss risks and potential complications associated with central venous access such as bleeding, thrombosis and infections. Patient access to ECP sites may be somewhat limited, though it is estimated that over 200 locations (mostly at transplant or academic medical centers) may be available worldwide [19]. Further expansion of ECP availability would help GVHD patients who are refractory to corticosteroids and in need of additional therapy.

The exact mechanism by which ECP induces tolerance in patients with GVHD remains an open question. There are several observations that may help to grasp current understanding of immunologic mechanisms of ECP. Data suggest that ECP-induced T-cell tolerance seems to depend on T-cell apoptosis, the presence of CD11c+ monocytes and regulatory T-cell (T-reg) induction [44,45,46,47]. Hannani et al. also suggested that alloreactive T-cells derived from GVHD patients are particularly susceptible to the pro-apoptotic effects of ECP [48]. However, only a small fraction (likely 5 to 10%) of circulating monocytes are exposed to UVA irradiation during ECP procedure, and it is less likely that this mechanism alone would explain the tolerance induction by ECP [49]. Additional considerations may need to be given to ECP effects on DCs as well as interactions of T-cells and DCs implicating the complex interplay of immunologic cells in exerting tolerance induced by ECP. The role of B-cells in the development of GVHD has also been investigated further in the recent past. Emerging data suggest profoundly impaired B cell immunity in chronic GVHD patients and some studies found that markers of B-cell activation may correlate with ECP responses [50,51]. Further research is needed to elucidate the mechanisms of GVHD response following ECP, to address questions regarding the differential effects on specific organs, and to develop new methods to improve ECP treatment responses in non-cutaneous GVHD.

## 6. Conclusions

Steroid-refractory GVHD remains a major cause of disabling morbidity and mortality after allogeneic HCT. ECP is an attractive second- and third-line option for those patients with GVHD, but its use may be influenced by clinical experience, center preference and patient access due in part to the lack of high-quality randomized clinical trials comparing ECP with other modalities, and paucity of algorithms incorporating ECP over other therapeutic options. The current decisions to prescribe ECP are largely made on an individual patient basis. The procedure has an excellent safety profile and it would be an ideal option for those who are unable to tolerate higher doses of corticosteroids. Extensive literature, albeit mostly retrospective in nature, exists on the efficacy of ECP in GVHD, and clinicians should consider ECP early on as a promising therapeutic modality for those patients who require corticosteroids for the treatment of GVHD. The standardization of ECP treatment may be important in delivering consistent therapy and produce reliable outcomes. With emerging GVHD therapies modulating JAK-STAT and BTK pathways, the treatment options for GVHD patients are growing, but ECP would be a useful therapeutic armamentarium for those GVHD patients undergoing life-threatening medical conditions.

## Figures and Tables

**Table 1:** Selected studies evaluating extracorporeal photopheresis in acute graft-versus-host disease.

Author (Reference)	Sample Size	Study Type	Underlying Hematologic Conditions	ORR	CR	aGVHD Organ Specific Responses		
						Skin	Liver	GI
***Prospective studies***								
Greinix et al. [9]	59	phase II prospective trial	AML, ALL, CML, other			82%	61%	61%
Alousi et al. [10]	51	phase II, randomized, adaptive Bayesian design-based study (ECP + methylprednisolone vs. methylprednisolone)	AML/MDS and others			72%	Visceral organ aGVHD: 47%	Visceral organ aGVHD: 47%
***Retrospective studies***								
Garban et al. [11]	12	retrospective	AL, CML, MDS, MM, Fanconi’s anemia, solid tumor	75%				
Perfetti et al. [12]	23	retrospective	CML, AML, MM, MDS, MF, AA, Behcet syndrome		52%	CR 66%	CR 27%	CR 40%
Jagasia et al. [13]	57	retrospective comparative analysis (ECP vs. anti-cytokine therapy)	Not reported	66%	54%	≤stage 2: 70% stage 3–4: 57%	≤stage 2: 72% stage 3–4: 50%	≤stage 2: 77% stage 3–4: 54%
***Systematic reviews***								
Abu-Dalle et al. [14]	323 (9 studies)-54 patients analyzed for ORR	Systematic review	AL, AA, MDS, Thalassemia major, MM, CML, CLL, NHL, Fanconi’s anemia, solid tumor	69%		84%		65%

ORR: overall response rate; CR: complete response; PR: partial response; aGVHD: acute graft-versus-host disease; GI: gastrointestinal; AML: acute myeloid leukemia; ALL: acute lymphoblastic leukemia; CLL: chronic lymphocytic leukemia; CML: chronic myelogenous leukemia; NHL: Non-Hodgkin’s lymphoma; MM: multiple myeloma; AA: aplastic anemia; ECP: extracorporeal photopheresis; AL: acute leukemia; MDS: myelodysplastic syndrome; MF: myelofibrosis.

**Table 2:** Selected studies evaluating extracorporeal photopheresis in chronic graft-versus-host disease.

Author (Reference)	Sample Size	Study Type	Underlying Hematologic Conditions	ORR	CR	cGVHD Organ Specific Response rate					
						Skin	Mucosa	Liver	GI	Ocular	Lung
***Prospective Studies***											
Flowers et al. [21]	48	Phase II RCT	AML, ALL, CML, NHL, and others			40%	53%	29%		30%	
Greinix et al. [22]	29	Open-label crossover ECP study	AL, CL, HL, MDS	31%		31%	70%	50%	60%	27%	57%
Foss et al. [23]	25	Prospective	CML, NHL, CLL, AML, ALL	64%	64%	80%	24%		46%		
Seaton et al. [24]	28	Prospective	AML, ALL, MM, CML			53%	50%				
***Retrospective Studies***											
Couriel et al. [26]	71	Retrospective	ALL, AML/MDS, CLL, CML, MPD, lymphoma, AA, SCA, breast cancer	61%	20%	57%	78%	71%		67%	54%
Dignan et al. [27]	82	Retrospective	AL, MDS, HL, NHL, CLL, CML, MM	79%	7%	100%	91%				
Del Fante et al. [28]	102	Retrospective	HL, AML, ALL, CLL, CML, NHL, MM, MDS, PNH	81%	16%						
***Systematic Reviews***											
Abu-Dalle et al. [14]	87 patients (5 studies), pooled results	Systematic review	AL, AA, MDS, Thalassemia major, MM, CML, CLL, NHL, Fanconi’s anemia, solid tumor	64%	26%	71%	63%	58%	62%		15%
Malik et al. [29]	595 patients (18 studies), pooled results	Systematic review/ meta-analysis	Various diseases	64%	29%	74%	72%	68%	53%	60%	48%
***Consensus Statements***											
Pierelli et al. [17]	735 patients (23 studies)	Consensus statements	Various diseases			64%	47–57%	27%	57%		
Scarisbrick et al. [30]	521 (23 studies)	Consensus statements	Various diseases			Mean 68%	Mean 63%	Mean 63%			
Alfred et al. [19]	725 (27 studies)	Consensus statements	Various diseases	Mean 68%		Mean 74%	Mean 62%	Mean 62%	Mean 46%	Mean 60%	Mean 46%

ORR: overall response rate; CR: complete response; PR: partial response; cGVHD: chronic graft-versus-host disease; RCT: randomized controlled trial; HL: Hodgkin’s lymphoma; GI: gastrointestinal; AML: acute myeloid leukemia; ALL: acute lymphoblastic leukemia; CLL: chronic lymphocytic leukemia; CML: chronic myelogenous leukemia; NHL: Non-Hodgkin’s lymphoma; MM: multiple myeloma; AA: aplastic anemia; ARDS: adult respiratory distress syndrome; ECP: extracorporeal photopheresis; AL: acute leukemia; MDS: myelodysplastic syndrome; MPD: myeloproliferative disease; SCA: sickle cell anemia; PNH, paroxysmal nocturnal hemoglobinuria.

## References

[1] Copelan E.A. (2006). Hematopoietic stem-cell transplantation. N. Engl. J. Med..

[2] Anasetti C., Logan B.R., Lee S.J., Waller E.K., Weisdorf D.J., Wingard J.R., Cutler C.S., Westervelt P., Woolfrey A., Couban S. (2012). Peripheral-blood stem cells versus bone marrow from unrelated donors. N. Engl. J. Med..

[3] Gratwohl A., Pasquini M.C., Aljurf M., Atsuta Y., Baldomero H., Foeken L., Gratwohl M., Bouzas L.F., Confer D., Frauendorfer K. (2015). One million haemopoietic stem-cell transplants: A retrospective observational study. Lancet Haematol..

[4] Martin P.J., Rizzo J.D., Wingard J.R., Ballen K., Curtin P.T., Cutler C., Litzow M.R., Nieto Y., Savani B.N., Schriber J.R. (2012). First- and second-line systemic treatment of acute graft-versus-host disease: Recommendations of the American Society of Blood and Marrow Transplantation. Biol. Blood Marrow Transplant..

[5] Knobler R., Barr M.L., Couriel D.R., Ferrara J.L., French L.E., Jaksch P., Reinisch W., Rook A.H., Schwarz T., Greinix H. (2009). Extracorporeal photopheresis: Past, present, and future. J. Am. Acad. Dermatol..

[6] Hymes S.R., Morison W.L., Farmer E.R., Walters L.L., Tutschka P.J., Santos G.W. (1985). Methoxsalen and ultraviolet A radiation in treatment of chronic cutaneous graft-versus-host reaction. J. Am. Acad. Dermatol..

[7] Gorgun G., Miller K.B., Foss F.M. (2002). Immunologic mechanisms of extracorporeal photochemotherapy in chronic graft-versus-host disease. Blood.

[8] Bruserud Ø., Tvedt T.H., Paulsen P.Q., Ahmed A.B., Gedde-Dahl T., Tjønnfjord G.E., Slåstad H., Heldal D., Reikvam H. (2014). Extracorporeal photopheresis (photochemotherapy) in the treatment of acute and chronic graft versus host disease: Immunological mechanisms and the results from clinical studies. Cancer Immunol. Immunother..

[9] Greinix H.T., Knobler R.M., Worel N., Schneider B., Schneeberger A., Hoecker P., Mitterbauer M., Rabitsch W., Schulenburg A., Kalhs P. (2006). The effect of intensified extracorporeal photochemotherapy on long-term survival in patients with severe acute graft-versus-host disease. Haematologica.

[10] Alousi A.M., Bassett R., Chen J., Overman B.J., Hosing C.M., Popat U.R., Shpall E.J., Nieto Y., Qazilbash M.H., Khouri I.F. (2015). A Bayesian, Phase II Randomized Trial of Extracorporeal Photopheresis (ECP) Plus Steroids Versus Steroids-Alone in Patients with Newly Diagnosed Acute Graft Vs. Host Disease (GVHD): The Addition of ECP Improves Gvhd Response and the Ability to Taper Steroids. Blood.

[11] Garban F., Drillat P., Makowski C., Jacob M.C., Richard M.J., Favrot M., Sotto J.J., Bensa J.C., Cahn J.Y. (2005). Extracorporeal chemophototherapy for the treatment of graft-versus-host disease: Hematologic consequences of short-term, intensive courses. Haematologica.

[12] Perfetti P., Carlier P., Strada P., Gualandi F., Occhini D., Van Lint M.T., Ibatici A., Lamparelli T., Bruno B., Raiola A.M. (2008). Extracorporeal photopheresis for the treatment of steroid refractory acute GVHD. Bone Marrow Transplant..

[13] Jagasia M., Greinix H., Robin M., Das-Gupta E., Jacobs R., Savani B.N., Engelhardt B.G., Kassim A., Worel N., Knobler R. (2013). Extracorporeal photopheresis versus anticytokine therapy as a second-line treatment for steroid-refractory acute GVHD: A multicenter comparative analysis. Biol. Blood Marrow Transplant..

[14] Abu-Dalle I., Reljic T., Nishihori T., Antar A., Bazarbachi A., Djulbegovic B., Kumar A., Kharfan-Dabaja M.A. (2014). Extracorporeal photopheresis in steroid-refractory acute or chronic graft-versus-host disease: Results of a systematic review of prospective studies. Biol. Blood Marrow Transplant..

[15] Messina C., Locatelli F., Lanino E., Uderzo C., Zacchello G., Cesaro S., Pillon M., Perotti C., Del Fante C., Faraci M. (2003). Extracorporeal photochemotherapy for paediatric patients with graft-versus-host disease after haematopoietic stem cell transplantation. Br. J. Haematol..

[16] Dignan F.L., Clark A., Amrolia P., Cornish J., Jackson G., Mahendra P., Scarisbrick J.J., Taylor P.C., Hadzic N., Shaw B.E. (2012). Diagnosis and management of acute graft-versus-host disease. Br. J. Haematol..

[17] Pierelli L., Perseghin P., Marchetti M., Messina C., Perotti C., Mazzoni A., Bacigalupo A., Locatelli F., Carlier P., Bosi A. (2013). Extracorporeal photopheresis for the treatment of acute and chronic graft-versus-host disease in adults and children: Best practice recommendations from an Italian Society of Hemapheresis and Cell Manipulation (SIdEM) and Italian Group for Bone Marrow Transplantation (GITMO) consensus process. Transfusion.

[18] Knobler R., Berlin G., Calzavara-Pinton P., Greinix H., Jaksch P., Laroche L., Ludvigsson J., Quaglino P., Reinisch W., Scarisbrick J. (2014). Guidelines on the use of extracorporeal photopheresis. J. Eur. Acad. Dermatol. Venereol..

[19] Alfred A., Taylor P.C., Dignan F., El-Ghariani K., Griffin J., Gennery A.R., Bonney D., Das-Gupta E., Lawson S., Malladi R.K. (2017). The role of extracorporeal photopheresis in the management of cutaneous T-cell lymphoma, graft-versus-host disease and organ transplant rejection: A consensus statement update from the UK Photopheresis Society. Br. J. Haematol..

[20] Das-Gupta E., Dignan F., Shaw B., Raj K., Malladi R., Gennery A., Bonney D., Taylor P., Scarisbrick J. (2014). Extracorporeal photopheresis for treatment of adults and children with acute GVHD: UK consensus statement and review of published literature. Bone Marrow Transplant..

[21] Rossetti F., Zulian F., Dall’Amico R., Messina C., Montini G., Zacchello F. (1995). Extracorporeal photochemotherapy as single therapy for extensive, cutaneous, chronic graft-versus-host disease. Transplantation.

[22] Rossetti F., Dall’Amico R., Crovetti G., Messina C., Montini G., Dini G., Locatelli F., Argiolu F., Miniero R., Zacchello G. (1996). Extracorporeal photochemotherapy for the treatment of graft-versus-host disease. Bone Marrow Transplant..

[23] Dall’Amico R., Rossetti F., Zulian F., Montini G., Murer L., Andreetta B., Messina C., Baraldi E., Montesco M.C., Dini G. (1997). Photopheresis in paediatric patients with drug-resistant chronic graft-versus-host disease. Br. J. Haematol..

[24] Greinix H.T., Volc-Platzer B., Rabitsch W., Gmeinhart B., Guevara-Pineda C., Kalhs P., Krutmann J., Hönigsmann H., Ciovica M., Knobler R.M. (1998). Successful use of extracorporeal photochemotherapy in the treatment of severe acute and chronic graft-versus-host disease. Blood.

[25] Smith E.P., Sniecinski I., Dagis A.C., Parker P.M., Snyder D.S., Stein A.S., Nademanee A., O’Donnell M.R., Molina A., Schmidt G.M. (1998). Extracorporeal photochemotherapy for treatment of drug-resistant graft-vs.-host disease. Biol. Blood Marrow Transplant..

[26] Flowers M.E., Apperley J.F., van Besien K., Elmaagacli A., Grigg A., Reddy V., Bacigalupo A., Kolb H.J., Bouzas L., Michallet M. (2008). A multicenter prospective phase 2 randomized study of extracorporeal photopheresis for treatment of chronic graft-versus-host disease. Blood.

[27] Greinix H.T., van Besien K., Elmaagacli A.H., Hillen U., Grigg A., Knobler R., Parenti D., Reddy V., Theunissen K., Michallet M. (2011). Progressive improvement in cutaneous and extracutaneous chronic graft-versus-host disease after a 24-week course of extracorporeal photopheresis--results of a crossover randomized study. Biol. Blood Marrow Transplant..

[28] Foss F.M., DiVenuti G.M., Chin K., Sprague K., Grodman H., Klein A., Chan G., Stiffler K., Miller K.B. (2005). Prospective study of extracorporeal photopheresis in steroid-refractory or steroid-resistant extensive chronic graft-versus-host disease: Analysis of response and survival incorporating prognostic factors. Bone Marrow Transplant..

[29] Seaton E.D., Szydlo R.M., Kanfer E., Apperley J.F., Russell-Jones R. (2003). Influence of extracorporeal photopheresis on clinical and laboratory parameters in chronic graft-versus-host disease and analysis of predictors of response. Blood.

[30] Rubegni P., Cuccia A., Sbano P., Cevenini G., Carcagnì M.R., D’Ascenzo G., De Aloe G., Guidi S., Guglielmelli P., Guglielmetti P. (2005). Role of extracorporeal photochemotherapy in patients with refractory chronic graft-versus-host disease. Br. J. Haematol..

[31] Couriel D.R., Hosing C., Saliba R., Shpall E.J., Anderlini P., Rhodes B., Smith V., Khouri I., Giralt S., de Lima M. (2006). Extracorporeal photochemotherapy for the treatment of steroid-resistant chronic GVHD. Blood.

[32] Dignan F.L., Greenblatt D., Cox M., Cavenagh J., Oakervee H., Apperley J.F., Fielding A.K., Pagliuca A., Mufti G., Raj K. (2012). Efficacy of bimonthly extracorporeal photopheresis in refractory chronic mucocutaneous GVHD. Bone Marrow Transplant..

[33] Del Fante C., Scudeller L., Viarengo G., Bernasconi P., Perotti C. (2012). Response and survival of patients with chronic graft-versus-host disease treated by extracorporeal photochemotherapy: A retrospective study according to classical and National Institutes of Health classifications. Transfusion.

[34] Malik M.I., Litzow M., Hogan W., Patnaik M., Murad M.H., Prokop L.J., Winters J.L., Hashmi S. (2014). Extracorporeal photopheresis for chronic graft-versus-host disease: A systematic review and meta-analysis. Blood Res..

[35] Scarisbrick J.J., Taylor P., Holtick U., Makar Y., Douglas K., Berlin G., Juvonen E., Marshall S., Group P.E. (2008). U.K. consensus statement on the use of extracorporeal photopheresis for treatment of cutaneous T-cell lymphoma and chronic graft-versus-host disease. Br. J. Dermatol..

[36] Dignan F.L., Amrolia P., Clark A., Cornish J., Jackson G., Mahendra P., Scarisbrick J.J., Taylor P.C., Shaw B.E., Potter M.N. (2012). Diagnosis and management of chronic graft-versus-host disease. Br. J. Haematol..

[37] Marks C., Stadler M., Häusermann P., Wolff D., Buchholz S., Stary G., Lee S., Lawitschka A., Bertz H. (2011). German-Austrian-Swiss Consensus Conference on clinical practice in chronic graft-versus-host disease (GVHD): Guidance for supportive therapy of chronic cutaneous and musculoskeletal GVHD. Br. J. Dermatol..

[38] Wolff D., Schleuning M., von Harsdorf S., Bacher U., Gerbitz A., Stadler M., Ayuk F., Kiani A., Schwerdtfeger R., Vogelsang G.B. (2011). Consensus Conference on Clinical Practice in Chronic GVHD: Second-Line Treatment of Chronic Graft-versus-Host Disease. Biol. Blood Marrow Transplant..

[39] Apisarnthanarax N., Donato M., Körbling M., Couriel D., Gajewski J., Giralt S., Khouri I., Hosing C., Champlin R., Duvic M. (2003). Extracorporeal photopheresis therapy in the management of steroid-refractory or steroid-dependent cutaneous chronic graft-versus-host disease after allogeneic stem cell transplantation: Feasibility and results. Bone Marrow Transplant..

[40] Ussowicz M., Musiał J., Mielcarek M., Tomaszewska A., Nasiłowska-Adamska B., Kałwak K., Gorczyńska E., Mariańska B., Chybicka A. (2013). Steroid-sparing effect of extracorporeal photopheresis in the therapy of graft-versus-host disease after allogeneic hematopoietic stem cell transplantation. Transplant. Proc..

[41] Ruutu T., Gratwohl A., de Witte T., Afanasyev B., Apperley J., Bacigalupo A., Dazzi F., Dreger P., Duarte R., Finke J. (2014). Prophylaxis and treatment of GVHD: EBMT-ELN working group recommendations for a standardized practice. Bone Marrow Transplant..

[42] Perotti C., Torretta L., Viarengo G., Roveda L., Bernuzzi S., Carbone S., Del Fante C., La Torre R., Locatelli F., Bonetti F. (1999). Feasibility and safety of a new technique of extracorporeal photochemotherapy: Experience of 240 procedures. Haematologica.

[43] Perotti C., Del Fante C., Tinelli C., Viarengo G., Scudeller L., Zecca M., Locatelli F., Salvaneschi L. (2010). Extracorporeal photochemotherapy in graft-versus-host disease: A longitudinal study on factors influencing the response and survival in pediatric patients. Transfusion.

[44] Maeda A. (2009). Extracorporeal photochemotherapy. J. Dermatol. Sci..

[45] Maeda A., Schwarz A., Kernebeck K., Gross N., Aragane Y., Peritt D., Schwarz T. (2005). Intravenous infusion of syngeneic apoptotic cells by photopheresis induces antigen-specific regulatory T cells. J. Immunol..

[46] Aubin F., Mousson C. (2004). Ultraviolet light-induced regulatory (suppressor) T cells: An approach for promoting induction of operational allograft tolerance?. Transplantation.

[47] Gatza E., Rogers C.E., Clouthier S.G., Lowler K.P., Tawara I., Liu C., Reddy P., Ferrara J.L. (2008). Extracorporeal photopheresis reverses experimental graft-versus-host disease through regulatory T cells. Blood.

[48] Hannani D., Merlin E., Gabert F., Laurin D., Deméocq F., Chaperot L., Kanold J., Plumas J. (2010). Photochemotherapy induces a faster apoptosis of alloreactive activated T cells than of nonalloreactive resting T cells in graft versus host disease. Transplantation.

[49] Goussetis E., Varela I., Tsirigotis P. (2012). Update on the mechanism of action and on clinical efficacy of extracorporeal photopheresis in the treatment of acute and chronic graft versus host disease in children. Transfus. Apher. Sci..

[50] Kuzmina Z., Greinix H.T., Knobler R., Worel N., Kouba M., Weigl R., Körmöczi U., Rottal A., Pohlreich D., Zielinski C. (2009). Proportions of immature CD19+CD21- B lymphocytes predict the response to extracorporeal photopheresis in patients with chronic graft-versus-host disease. Blood.

[51] Whittle R., Taylor P.C. (2011). Circulating B-cell activating factor level predicts clinical response of chronic graft-versus-host disease to extracorporeal photopheresis. Blood.

